# A tailored distractor-assisted percutaneous minimally invasive treatment for Sanders II and III calcaneus fractures: a consecutive cohort study with medium-term results

**DOI:** 10.3389/fsurg.2025.1599356

**Published:** 2025-08-01

**Authors:** Xiong Liao, Jianliang Deng, Wei Liu, Di You

**Affiliations:** Department of Orthopedic Trauma, The Affiliated Changsha Central Hospital (Changsha Central Hospital), Hengyang Medical School, University of South China, Changsha, China

**Keywords:** calcaneus fracture, calcaneal distractor, foot surgery, fracture reduction, minimally invasive surgery

## Abstract

**Background:**

The treatment strategy of displaced intra-articular calcaneal fractures (DIACFs) remains challenging. While the operation techniques vary widely, the efforts is now on the optimization of surgical techniques to better DIACFs management. This study aimed to introduce a tailored distractor-assisted percutaneous minimally invasive surgery (MIS) and reported its medium-term outcomes in patients with Sanders II and III calcaneus fractures.

**Methods:**

63 cases (63 feet) of DIACFs, subjected to a tailored distractor-assisted MIS in our orthopedic department were retrospectively analyzed. The medical records and radiological measurements were retrieved for efficiency evaluation. At the last follow-up, the American Orthopaedic Foot & Ankle Society (AOFAS) ankle-hind foot score and Visual Analog Scale (VAS) score were used to evaluate the functional rehabilitation. Postoperative complications were also recorded.

**Results:**

All feet (39 Sanders type II fractures and 24 Sanders type III) successfully achieved fracture reduction with the interval between injury to operation average 1.3 days, the duration of surgery average 40.1 min, and the hospital stay average 4.9 days. Radiographic measurements revealed significant differences between pre-operation and post-operation in calcaneal height and width, so as to Bohler's angle and Gissane angle (*p* < 0.05, respectively). Anatomical or near-anatomical realignment of the posterior subtalar articular surfaces were achieved in all cases. At the last follow-up, the AOFAS and the VAS score was average 81.4 and 1.3 points, both significantly improved from that of pre-operation (*p* < 0.05, respectively). Four feet (6.3%) encountered postoperative complications.

**Conclusion:**

Application of the tailored calcaneal distractor in MIS for Sanders type II and III calcaneal fractures has demonstrated advantages in effectively manipulating fracture reduction and yielding favorable clinical outcomes. Further cohort study is required to clarify its clinical significance vs. other techniques.

## Introduction

Calcaneal fractures, common foot injuries often resulting from high-energy trauma, presents great clinical challenges due to their complex anatomical structure and post-traumatic sequelae (1). Sanders (2) developed a computed tomography–based classification to assist decision-makings and preoperative planning. Conservative treatment, such as using Plaster-of Paris, might be considered in fractures with little displacement (Sanders type I) or compromised soft-tissues. Surgical intervention is typically preferred for displaced intra-articular calcaneus fractures (DIACFs), which accounts for approximate 75% of calcaneal fractures (3, 4). In recent years, various minimally invasive techniques were developed and applied to improve DIACFs treatment. In comparison with traditional open surgery, minimally invasive surgery (MIS) showed advantages in minimizing iatrogenic tissue disturbance and incision-related complications while pursuing fracture reduction and fixation (5). However, the MIS approach is difficult to realize adequate fracture reduction and posterior facet realignment because of a limited visualization and percutaneous performance, leading to ongoing debate in this field (6, 7).

**Figure 1:**
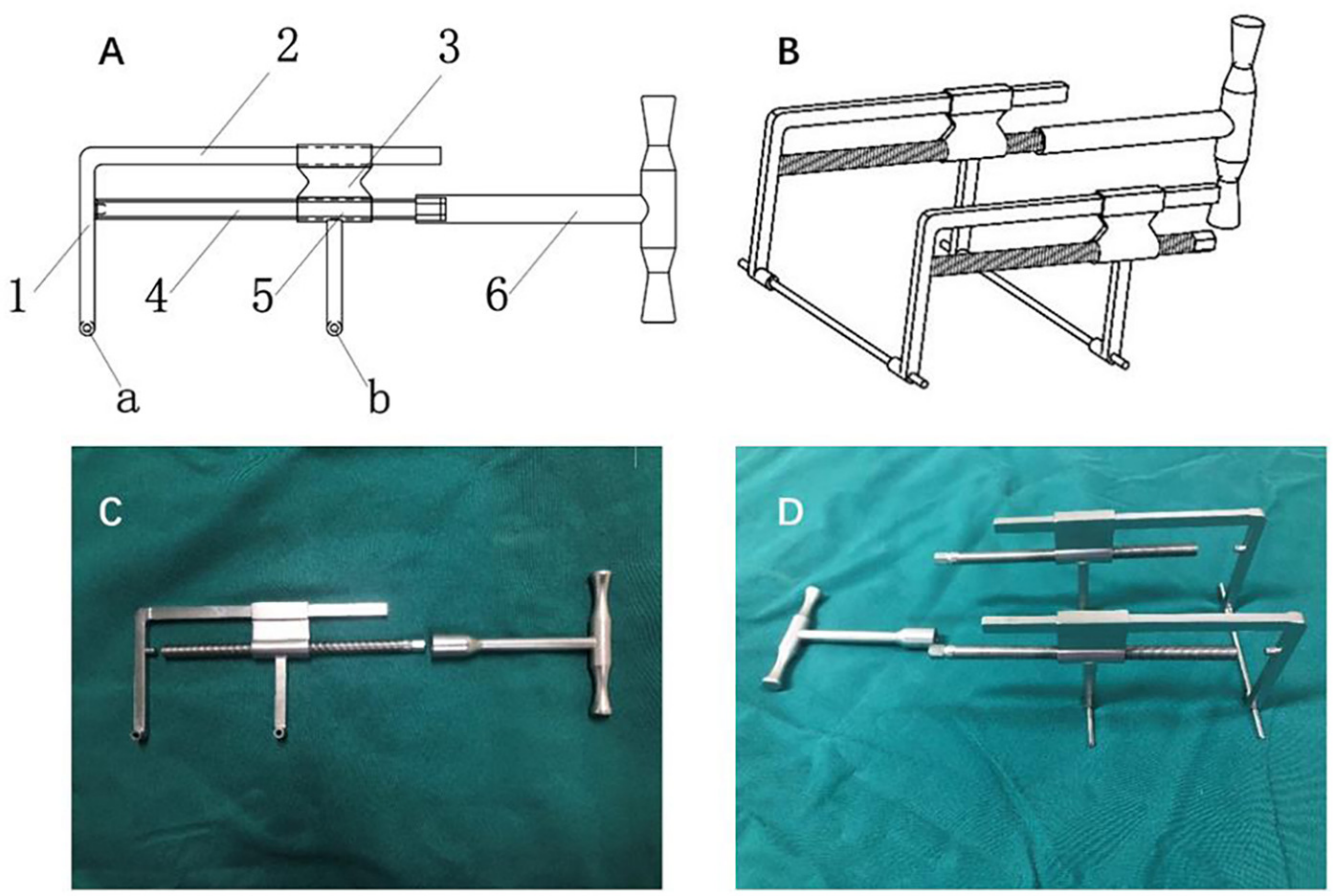
The novel self-constructed calcaneal distractor: **(A,B)** are schematic blueprint-a and b are traction pin holes, 1 is the fixed supported rod 2, is the connecting rod, 3 is the sliding support rod, 4 is the threaded supported rod, 5 is the threaded sleeve, and 6 is the “T” handle; **(C,D)** are product photos.

Calcaneal distractor as the imperative assistive instruments plays an key role in MIS for DIACFs and, to a certain extent, closely determines the surgical outcomes (8). To facilitate fracture distraction and improve surgical outcomes, we designed a novel calcaneal distractor (National Invention Patent) based on the principle of ligamentotaxis (9). The tailored new calcaneal distractor (Figure 1) basically consists of two traction pins, two distraction assemblies, and a “T”-shaped handle. Each distraction assembly includes a fixed support rod, a connecting rod, a sliding support rod, and a threaded support rod, with a threaded sleeve in the sliding support rod. By rotating the “T” handle, the threaded support rod is mechanically driven to move forward in the threaded sleeve. When the front end of the threaded support rod contacts the fixed support rod, based on the principle of action and reaction, the sliding support rod is pulled away from the fixed support rod, bilaterally achieving a multi-dimensional distraction.

At our institution, this tailored distractor was frequently applied to assist MIS for DIACFs. This paper aimed to assess its efficiency of the tailored calcaneal distractor in percutaneous minimally invasive treatment for Sanders II and III type calcaneus fractures and report the surgical outcomes during a 2-year follow-up.

## Methods and materials

### Patient cohort

After admission in the orthopedics department of our hospital, routine lateral, axial x-rays, as well as three-dimensional CT scans of the calcaneus, were performed to confirm the DIACFs' diagnosis, classification, and fracture displacement patterns, thereby supporting the medical decision-makings. On our schedule, after completing the preoperative preparation and evaluation, a tailored calcaneal distractor-assisted percutaneous minimally invasive surgery was performed as quick as possible irrespective of the “wrinkle sign”.

Inclusion Criteria: closed Sanders type II or III calcaneal fractures; unilateral isolated calcaneal fractures; age ≥18 years; complete follow-up data of 24 months or more. Exclusion Criteria: open calcaneal fractures; concomitant injuries requiring other surgical operations; Previous fracture or surgical history of the calcaneus; pathological fractures; incomplete follow-up data or follow-up period less than 24 months.

From March 2020 to August 2022, 63 patients (63 feet) with calcaneal fractures were included in this study per the eligibility criteria. The demographic characteristics were retrieved from the electronic records and summarized in Table 1. This study was approved by the Medical Ethics Committee of our hospital (2021-S0052), and all patients signed the informed consent form. All surgeries were performed by the same experienced surgeon.

**Table 1:** Cohort demographic data in this study (*n* = 63).

Characteristics	Number of patients *N* (%)
Age (average, rang)	36 years, 19–69 years
Gender	
Male	52 (82.5)
Female	11 (17.5)
Side	
Left	35 (55.6)
Right	28 (44.4)
Mechanism of injury	
Fall from a height	46 (73.0)
Traffic trauma	17 (27.0)
Sanders classification	
Type II	39 (61.9)
Type III	24 (38.1)
Clinic data*	
Interval between injury and surgery (days)	1.3 ± 0.7
Time of operation (minutes)	40.1 ± 8.4
The duration of hospital stay (days)	4.9 ± 1.8

* Data presented as mean ± standard deviation.

### Surgical procedures and technique notes

**Figure 2:**
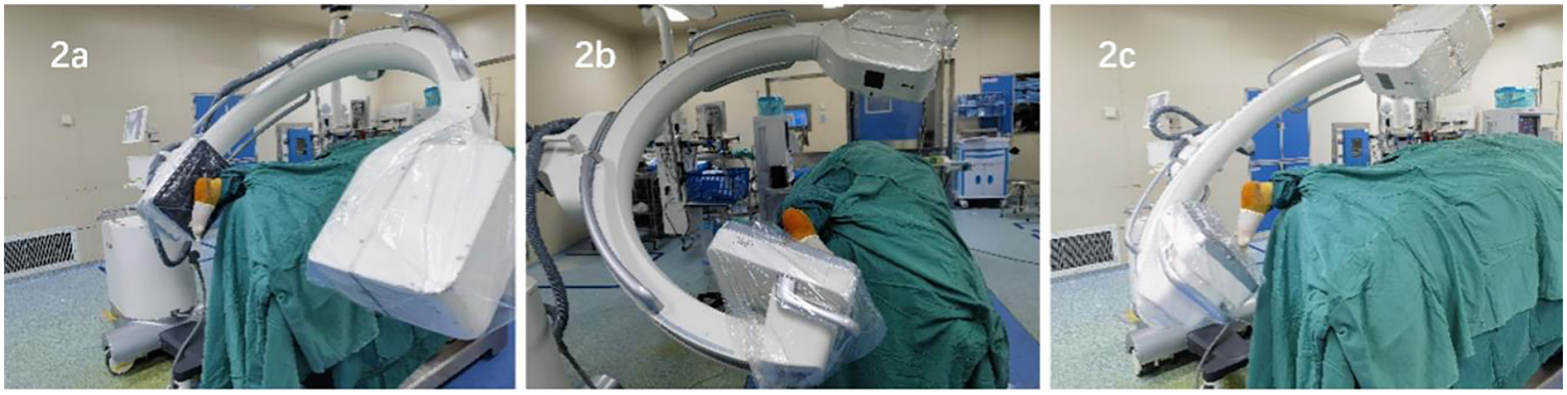
Photos pf patient positioning and placement of the C-arm x-ray machine **(a)** lateral view of the calcaneus under fluoroscopy, **(b)** broden view of the calcaneus under fluoroscopy, **(c)** axial view of the calcaneus under fluoroscopy.

Patient Positioning and C-arm Machine Placement: The patient is placed in the prone position after anesthesia with the affected leg on top. The C-arm is fixed at a forward tilt of 135 degrees. A lateral view of the calcaneus is obtained when the C-arm is rotated to 90° horizontally (Figure 2a), a Broden view when rotated to 35° horizontally with the affected foot positioned in internal rotation (Figure 2b), and an axial view when rotated to 10° horizontally (Figure 2c). Following this pattern, it is easy to obtain and quickly switch between the three different fluoroscopic views during operation. The patient's position and the placement of the C-arm x-ray machine must be confirmed before draping. Obtaining good fluoroscopic views is crucial for subsequent operation.

**Figure 3:**
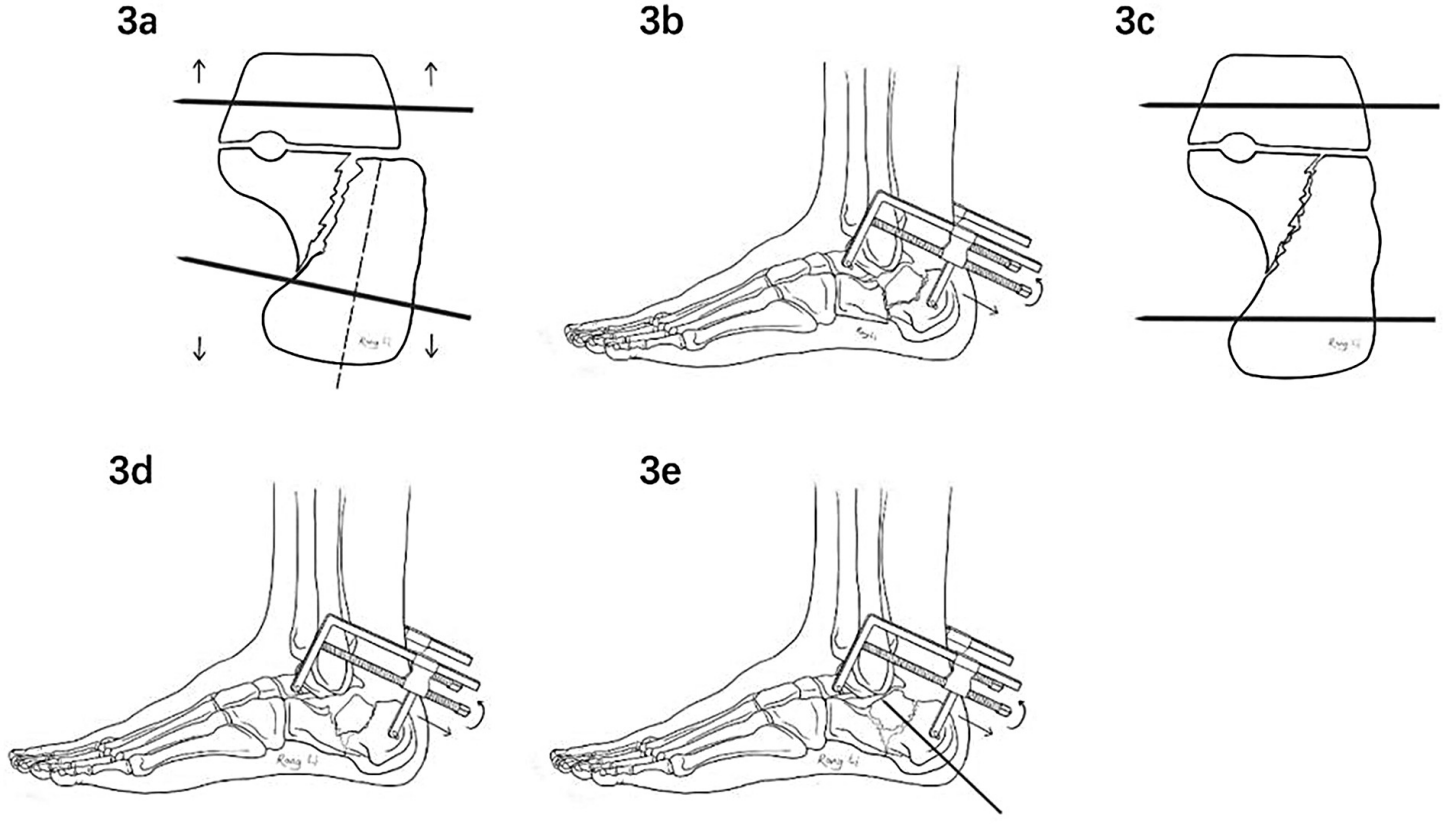
Flow diagramm of manipulating the modified calcaneal distractor: **(a)** the kirschner wire passing through the posterior calcaneal tuberosity must be perpendicular to the long axis of the calcaneus; **(b)** attach the medial and lateral distractor components to the ends of the kirschner wire, completing the installation of the distractor; **(c,d)** through closed distraction, the length and height of the calcaneus can be quickly restored, correcting inversion and eversion deformities; **(e)** insert a kirschner wire percutaneously to lever and reduce the posterior subtalar joint fracture fragment.

Installation of the tailored Calcaneal Distractor: Use fluoroscopy to locate two points on the long axis of the calcaneus, specifically the center of the talar neck and the center of the calcaneal tuberosity, followed by inserting two 3.0 mm Kirschner wires percutaneously from the two located points in an outside-in direction. The Kirschner wire passing through the talar neck anteriorly should be inserted horizontally, while the wire passing through the calcaneal tuberosity posteriorly should be inserted perpendicularly to the long axis of the calcaneus (adjust under axial fluoroscopic monitoring, Figure 3a). Attach the medial and lateral distractor components to the ends of the Kirschner wires, respectively (Figure 3b).

Percutaneous Fracture Reduction: According to the degree of inversion, eversion, and shortening of the calcaneus, rotate the “T”-shaped handle to sequentially distract the medial and lateral components to varying degrees of wide range in a manner of synergistic manipulation bilaterally. Based on the principle of ligamentotaxis, the length, height, and varus or vaglus deformities of the calcaneus can be handily restored (Figures 3c,d), followed by checking the alignment of the posterior calcaneal tuberosity with the sustentaculum tali and the medial wall in the axial view. Once above restoration completed, the neutral triangle area of the calcaneal body becomes empty. At this point, the surgeon performs percutaneous compression on the lateral side of the affected hind-foot to quickly restore the width of the calcaneus. When it comes to levering, referring to fluoroscopic images in the lateral, axial and Broden's views of the calcaneus, insert a Kirschner wire percutaneously into the displaced joint fracture fragment and leverage it in the direction opposite to the fracture displacement to restore the posterior subtalar joint surface, while reducing the displaced fragments (Figure 3e). Once reduction is satisfactory, temporarily fix it with a percutaneous Kirschner wire.

Percutaneous Screw Fixation: Ahead of screw insertion, distribution and configuration of the screws fixation have to be conceived of forming a framework structure around the neutral triangle of the calcaneus typically via three 5.2 mm diameter full-threaded cannulated screws. One to two 4.0 mm diameter partially-threaded cannulated lag screws are typically inserted transversely from the lateral side into the sustentaculum tali along the guide pins sequentially to compress and fix the posterior subtalar joint surface. This screw distribution and configuration form a framework structure to provide reliable initial stability conducive to fracture healing. The incisions were sutured in single layer fashion.

### Postoperative management and follow-up

Patients were scheduled for a follow-up of 24 months post-surgery for guidance on functional exercises and clinical outcome assessment. No cast was used postoperatively. On the first day after surgery, patients were encouraged to perform active and passive ankle and foot exercises. From the 6th week post-surgery, patients gradually began partial weight-bearing walking on the affected foot with crutch assistance, followed by full weight-bearing walking without crutches from the 9th to the 12th week post-surgery based on x-ray evidence of fracture healing. Lateral and axial x-rays and three-dimensional CT scans of the calcaneus were performed on the first day postoperatively and at the final follow-up to measure the length, width, and height of the calcaneus, Böhler's angle, and Gissane's angle. The reduction quality of the posterior subtalar joint surface were judged based on coronal CT images per Sanders standard (10). At the final follow-up, the American Orthopaedic Foot and Ankle Society (AOFAS) ankle-hind foot score (7) was used to evaluate the hind foot function rehabilitation, and the Visual Analog Scale (VAS) to assess pain symptom.

### Statistical analysis

Statistical analyses were performed using SPSS 22.0 software. The Shapiro–Wilk test was used to check the normality of the data. Normally distributed data with homogeneity of variance were expressed as mean ± standard deviation (*x* ± *s*). The radiographic parameters of the calcaneus and functional outcomes were compared by paired *t*-tests or independent *t*-tests, wherever appropriate. The statistical analysis was performed by two listed authors independently. A *P*-value <0.05 was set to indicate statistical significance.

## Results

Perioperatively, all feet (39 Sanders type II and 24 Sanders type III fractures) successfully achieved fracture reduction with the interval between injury to operation average 1.3 days, the duration of operation average 40.1 min, and the hospital stay average 4.9 days (Table 1).

Radiographic measurements revealed significant differences between pre-operation and post-operation in calcaneus width (43.7 ± 4.7 vs. 33.2 ± 1.9, *p* < 0.05) and height (37.0 ± 1.9 vs. 43.2 ± 2.0, *p* < 0.05), while no differences between post-operation and the normal side (*p* > 0.05). The length was comparable between pre-operation, post-operation, and the normal side (*p* > 0.05) (Table 2). Böhler's angle at the final follow-up was significantly improved from pre-operation measurements (34.0 ± 2.6 vs. 11.1 ± 2.1, *p* < 0.05), so as to Gissane's angle (122.7 ± 3.8 vs. 99.8 ± 3.9, *p* < 0.05), while no significant differences were detected between the post-operative and the contralateral side Bohler's angle and Gissane's angle (*p* > 0.05, respectively) (Table 2).

**Table 2:** Radiographic outcomes (*n* = 63).

Measurements (*Mean* *±* *SD*)	Length (mm)	Width (mm)	Height (mm)	Gissane's angle (°)	Bohler's angle (°)
Pre-operation	70.6 ± 1.5	43.7 ± 4.7	37.0 ± 1.9	99.8 ± 3.9	11.1 ± 2.1
Post-operation	70.8 ± 1.4	33.2 ± 1.9	43.2 ± 2.0	122.7 ± 3.8	34.0 ± 2.6
Normal side	71.1 ± 1.8	32.8 ± 1.8	43.1 ± 2.2	123.5 ± 3.3	34.3 ± 3.8
*p* Value (Pre- vs Post-operation)	*t* = −0.511	*t* = 15.434	*t* = −42.663	*t* = −36.288	*t* = −58.693
	*p* = 0.611	***p*** **=** **0.000**	***p*** **=** **0.000**	***p*** **=** **0.000**	***p*** **=** **0.000**
*p* Value (Post-operation vs Normal)	*t* = −1.924	*t* = 1.152	*t* = 0.297	*t* = −1.530	*t* = −0.373
	*p* = 0.059	*p* = 0.254	*p* = 0.767	*p* = 0.131	*p* = 0.711

Bold values indicate statistical significance based on paired *t*-test.

At the last follow-up, the AOFAS score was significantly improved from preoperation (81.4 ± 7.6 vs. 51.2 ± 3.1, *p* < 0.05), with classification distributed as 39 excellent, 21 good, and 3 fair classification, resulting in an excellent and good rate of 95.2%. The VAS score was significantly decreased from preoperation (1.3 ± 1.0 vs. 7.3 ± 1.4, *p* < 0.05) at the last follow-up (Table 3).

**Table 3:** AOFAS and VAS scores: pre-operation vs. the last follow-up.

Scores (*Mean* ± *SD*)	AOFAS	VAS
Pre-operation	51.2 ± 3.1	7.3 ± 1.4
Last follow-up	81.4 ± 7.6	1.3 ± 1.0
*p* Value (Pre-operation vs. The last follow-up)	*t* = −36.659	*t* = 26.001
	***p*** **=** **0.000**	***p*** **=** **0.000**

AOFAS, American Orthopaedic Foot & Ankle Society; VAS, visual analog scores.

Bold values indicate statistical significance based on paired *t*-test.

Additionally, the reduction quality of the posterior subtalar joint surface, as evidenced on CT images, were confirmed as Anatomical or near-anatomical realignment. Four feet (6.34%) encountered postoperative complications: one case got caught in transient symptoms of sural nerve injury, one case encountered a screw rupture 2 months after surgery without further detrimental effects, and two cases developed degenerative changes of the subtalar joint.

**Figure 4:**
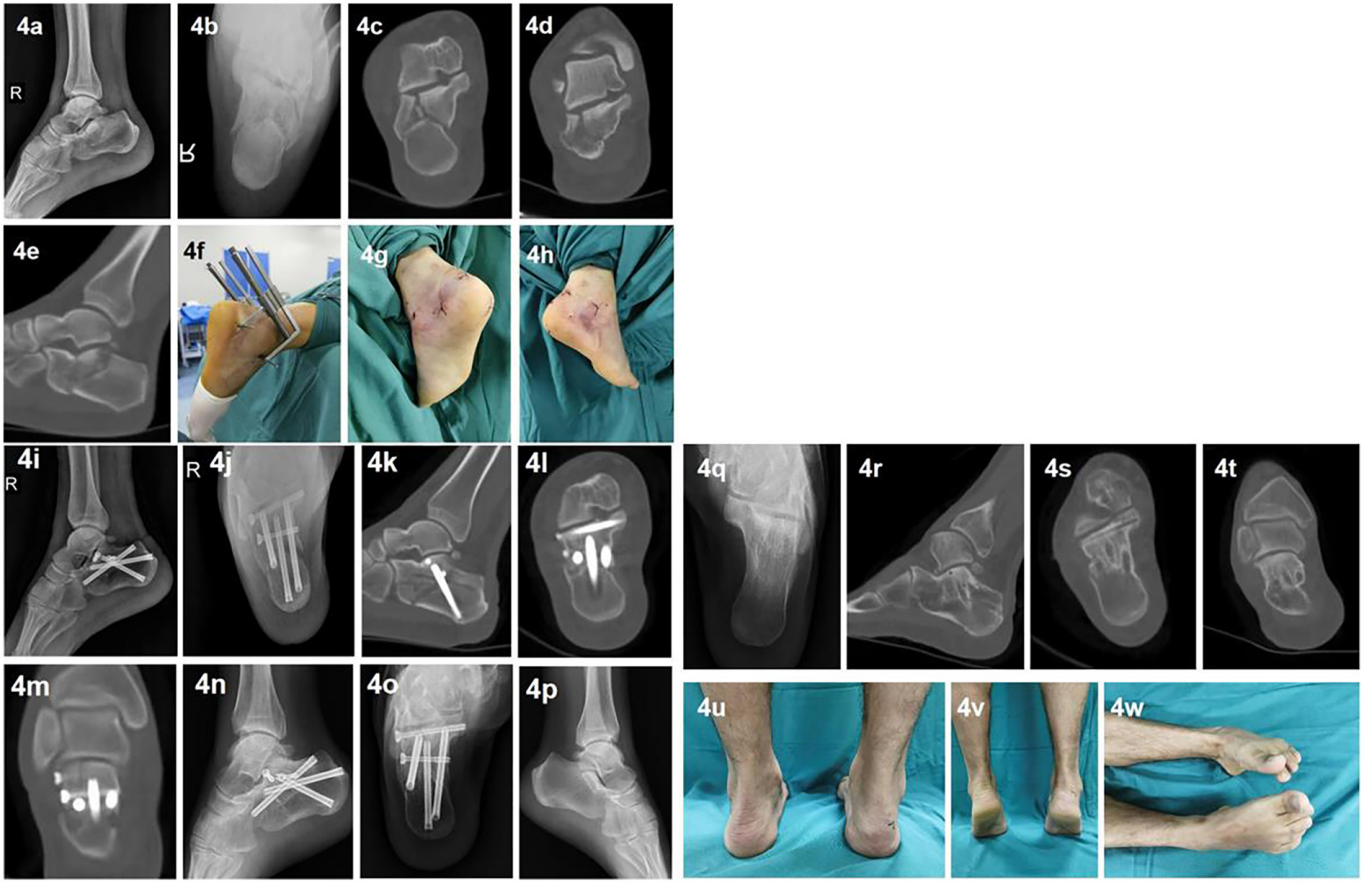
Illustration case: A 42-year-old male patient with a right calcaneal fracture (Sanders type IIa) due to a fall from a height. Preoperative lateral and axial x-rays **(a,b)** and axial, coronal, and sagittal CT scans **(c–e)** show shortening, height loss, and widening of the calcaneus, with significant collapse and displacement of the posterior subtalar joint surface and varus deformity. On the day of admission, the patient underwent closed fracture reduction and percutaneous cannulated screw fixation assisted by the modified calcaneal distractor **(f)** The operation took 45 min ending with single layer suture **(g,h)**. Postoperative lateral and axial x-rays **(i,j)** and sagittal, axial, and coronal CT scans **(k–m)** show complete restoration of the calcaneal length, width, and height, with complete correction of the collapse and displacement of the posterior subtalar joint surface, the joint surface fracture gap <1 mm, and complete correction of the varus deformity. The patient was discharged at 4th days after operation and went through a two-year event-free follow-up. 12 months postoperation, the lateral and axial x-rays show complete healing of the calcaneus **(n,o)**, with Bohlers's and Gissa's angle comparable to the normal side **(p,q)**. After removal of the internal fixation at 24 months postoperatively, CT scanns (1 − *t*) show no loss of reduction and no signs of subtalar arthritis. The final follow-up photos (u-w) show normal hindfoot alignment, no varus or valgus deformity, and good function of the ankle and subtalar joints. The AOFAS score was 98 points, and the VAS score was 0 points.

Illustration case Please see Figure 4.

## Discussion

The treatment of displaced intra-articular calcaneal fractures (DIACFs) presents a clinical dilemma where the optimal techniques remain debating (11, 12). Recently, minimally invasive surgery (MIS) reportedly had advantages in minimal invasion and lower risk of would-related complications over conventional open surgery or semi-closed reduction, such as sinus tarsi approach (STA) (13–15). However, less precise reduction of calcaneal fractures limited the application of MIS for DIACFs (3). Notably, the tailored distractor-assisted MIS described in this study demonstrated a balance between minimal invasion and adequate reduction and reliable fixation of calcaneal fractures via percutaneous manner. With all surgical steps handily reproducible and performed without exposing the surgeon to x-rays, our interpretation of this technique would greatly contribute to its widespread adoption in hospitals.

The primary goal of calcaneal fracture treatment is to achieve anatomy reduction and reliable fixation (16). Technically, percutaneous minimally invasive techniques for DIACFs imperatively require adjuvant tools to assist fracture distraction and fixation via percutaneous maneuver. In addition, the irregular shape of the calcaneus and the varied displacement patterns of the fractures render MIS more challenging (9). In such settings, many auxiliary devices have been successively innovated and applied in MIS for calcaneal fractures. Traditionally, Steinmann pins or Schanz screws were used to traverse the posterior calcaneal tuberosity, restoring the length of the calcaneus and correcting varus and valgus deformities through manual traction (17). The traction force is limited and cannot sustain the reduction, and surgeons often have to work directly under x-ray exposure. Driessen et al. (11) was among the first to use a three-point distractor to assist minimally invasive treatment of calcaneal fractures, a technique later adopted by other researchers. Despite some improvements, their distractor had a complex structure with inconvenient operablity. Zhao et al. (18) used a single Steinmann pin distractor placed on the lateral side of the affected foot, combined with a sinus tarsal incision, to treat calcaneal fractures. Such a distractor, which operates in an arc, is not fully effective in its distracting role, and a unilateral distractor might cause calcaneal varus or valgus deformities due to its eccentric traction force. Recognizing bilateral distractors superior to unilateral ones in correcting varus and valgus deformities, Dayton et al. (19) used an Ilizarov external fixator to assist in reduction, combined with bilateral small external fixators for percutaneous fixation of calcaneal fractures. This distractor is more complex and obstructs intraoperative fluoroscopy. Two-point distractor are generally adopted to bilaterally reduce calcaneus fracture ever after (20, 21). Although those MIS techniques vary widely, mal-reduction, no hand-free distraction, reduction loss, and occupation of operation space were the major drawbacks emerging in the former clinical practices.

Developed but different from the principle of ligamentotaxis, we designed a self-constructed calcaneal distractor with integrated structures to makes up for the shortcomings of former distractors. The tailored distractor we constructed harbors the following advancements and innovations (Please refer to Figures 1, 2): First, it has a simple structure, including two traction pins, two sets of retraction components, and a “T”-sharped handle. The traction pins are 3.0 mm diameter ordinary Kirschner wires, which are resistant to deformation ensuring effective retraction. The “T” handle is detachable, leaving more operating space. Second, it is easy to operate. The insertion points of the Kirschner wires are located at the center of the talar neck and the center of the calcaneal tuberosity, avoiding important blood vessels, nerves, and tendons. The two insertion points are on the long axis of the calcaneus, maximizing the effect of ligamentotaxis; by simply rotating the “T” handle, the fracture ends of the calcaneus can be effectively retracted mechanically. Third, it provides strong retraction force bilaterally and aids significantly in reduction. Once the retractor is installed, the medial and lateral retraction components can be adjusted to varying degrees per characteristic displacement patterns. The length and height of the calcaneus can be quickly restored, synergistically with the inversion and/or eversion deformities correction. Fourth, it can stably maintain the retracted state without obstructing fluoroscopy. Once the calcaneal fracture ends are retracted, enough sub-talar space is provided, facilitating the realignment of subtalar joint surface. Fifth, all operations can be performed without exposure to x-rays for operators. Given those characteristics, we hypothesized that our distractor is ideally modified to facilitate the percutaneous maneuver and improves surgical outcomes in DIACFs treatment.

As a result, all cases in the present study were successfully managed by our distractor-assisted reduction and percutaneous cannulated screw fixation. The tailored-assisted MIS showed advantages in the interval from injury to surgery, operation time, and the hospital stay when comparing with other reports regarding conventional STA or open approach reduction (22–24). Notably in our practice, the tailored distractor-assisted MIS are particularly appropriate for Sanders II and III calcaneus fractures earlier in the post injury period irrespective of the skin blister. As comparable with other minimally invasive techniques noted rare wound complications (6, 15, 17), our cases had not encountered any wound-related complications. Schepers (25) published a systemic review regarding sinus tarsi approach (STA) in DIACFs and found that wound complications were reported in all studies with a rate average 4.8%. A conspicuous advantage of MIS in comparison with conventional STA approach for DIACFs is the earlier surgical intervention and minimal invasion. The earlier intervention enables timely and effective fracture reduction, benefiting the subsequent rehabilitation progress (26–28). Additionally, the percutaneous approach results in less scar tissue formation around the ankle and subtalar joints, both contributing to the lower risk of developing wound-related complications and other degenerative changes of subtalar joint, which were common complications reported in other studies (29, 30). Judging from the surgical outcomes, our MIS strategy could effectively minimize iatrogenic tissue disturbance and incision-related complications while pursuing fracture reduction and fixation in comparison with traditional open or STA methods.

As discussed above, the current existing MIS techniques reflect a noteworthy drawback of inadequate reduction because of the limited visualization and indirect maneuver (31–33). Various imaging parameters of the calcaneus are available to evaluate the quality of fracture reduction, especially Gissane's angle and Böhler's angle, which are closely related to postoperative functional outcomes (34). Gavlik et al. reported reduction collapse or loss when using screws alone to fix calcaneal fractures, while this issue did not occur in our study (30). Recently, Zhang presented a T-handles rod distractor, screwed together with a cannulated screw, to assist reduction for the joint depression-type of calcaneal fractures via STA (35). They reported reduction failure because the distractor cannot hold the calcaneal tuberosity tightly or the inner rod could puncture the subtalar articular surface. In the present study, postoperative x-rays and CT scans showed complete restoration of the calcaneal length, width, and height, realignment of the collapse and displacement of the posterior subtalar joint surface, as well as complete correction of the varus or valgus deformity, both comparable with normal side indices. Adequate and precise fracture reduction achieved in this study could be partly explained by the tailored distractor providing powerful and multi-dimensional distraction force, which is outperform the AO two-point distractor offering retraction bilaterally (21). Furthermore, the technique underscored percutaneous distribution of cannulated screws. The screw placement configuration ensures a frame structure around the calcaneal “neutral triangle” to provide good initial stability conducive to fracture healing. Even in osteoporotic cases, our distractor seemed to be osteoporosis-friendly considering its structural features by distributing the force of distraction evenly via two 3.0 mm Kirschner wires and gently through bilateral threaded-rod adjustment, which definitely deserves future investigation to clarify this advantage in osteoporotic setting. Therefore, despite via percutaneous minimally invasive approach, our MIS strategy yielded comparable radiological outcomes and functional scores with conventional open or STA surgery (26, 36, 37) while outperforming other MIS techniques about this matter (4, 6, 20, 21). However, it should be cautious for utilizing this minimally invasive technique to treat Sander type IV fractures characterized by severe comminuted subtalar fragments. We have zero MIS experience dealing with Sanders type IV calcaneal fracture.

In conclusion, the tailored distractor demonstrated strong operability and effectiveness in fracture reduction. Subsequently, the distractor-assisted MIS for Sanders II and III calcaneus fractures realized adequate fracture reduction and reliable internal fixation, equivalent to conventional STA methods but a major previous limitations in other MIS techniques. As the implementation of ERAS (enhanced recovery after surgery) pathways developing (38), this study provides a promising therapeutic option for better DIACFs management. However, our study also has inherent limitations. As a retrospective study, it failed to set a control group, which compromised the interpretation of its clinical significance; Second, the follow-up period was short, making it difficult to evaluate long-term complications such as subtalar arthritis. Third, his study failed to interpret difference between Sanders II and III types because there were no enough cases to carry out subgroup analysis. Further randomized controlled studies with larger sample sizes and longer follow-up periods are needed.

## Conclusion

Application of the tailored calcaneal distractor in MIS for Sanders type II and III calcaneal fractures has demonstrated advantages in effectively manipulating fracture reduction and yielding favorable clinical outcomes. Further cohort study is required to clarify its clinical significance vs. other techniques.

## Data Availability

The raw data supporting the conclusions of this article will be made available by the authors, without undue reservation.
